# Reliability of Disk Diffusion Test Results for the Antimicrobial Susceptibility Testing of Nosocomial Gram-positive Microorganisms: Is E-test Method Better?

**Published:** 2012

**Authors:** Hossein Khalili, Rasool Soltani, Sorrosh Negahban, Alireza Abdollahi, Keirollah Gholami

**Affiliations:** a *DepartmentofClinicalPharmacy, FacultyofPharmacy, TehranUniversityofMedicalSciences, Tehran, Iran. *; b *DepartmentofClinicalPharmacy, FacultyofPharmacy, IsfahanUniversityofMedicalSciences, Isfahan, Iran*; c *MicrobiologyLaboratory, ImamKhomeiniHospital, Tehran, Iran. *; d *DepartmentofPathology, FacultyofMedicine, TehranUniversityofMedicalSciences, Tehran, Iran.*; e *DepartmentofClinicalPharmacy, FacultyofPharmacy, TehranUniversityofMedicalSciences, Tehran, Iran.*

**Keywords:** Gram-positive, Antimicrobial susceptibility test, Disk diffusion, E-test

## Abstract

Disk diffusion test is the usual applicable method for assessing the antimicrobial susceptibility pattern in most institutions and hospitals. The aim of this study was to determine the reliability of resistant-reported results of disk diffusion test for 6 routinely used antibiotics against Gram-positive microorganisms of nosocomial origin, using E-test method.

Over a 1-year period, clinical specimens (*e.g*. blood, tracheal secretions, wound secretions, urine, *etc*.) were obtained from hospitalized patients with defined nosocomial infection and were cultured. Isolated Gram-positive bacteria underwent disk diffusion test for cephalothin, oxacillin, clindamycin, ciprofloxacin, vancomycin, teicoplanin (only for Enterococci), and meropenem antibiotics. E-test method was performed for all isolates resistant or intermediately sensitive to the disks of any mentioned antibiotics.

Data showed compatible results of disk diffusion test with the results of E-test method for cephalothin, oxacillin, ciprofloxacin, vancomycin, and teicoplanin. None of ciprofloxacin- and vancomycin-resistant isolates in disk diffusion test showed sensitivity in E-test method. Significant differences between the results of disk diffusion and E-test methods were observed for clindamycin and meropenem against *S.aureus* (p = 0.01 and 0.04, respectively) and *Enterococcus spp* (p = 0.03 and 0.02, respectively).

In order to increase the reliability of antimicrobial susceptibility results, it is recommended to perform E-test for nosocomial Gram-positive microorganisms that show antibiotic resistance by disk diffusion test and it is more important for clindamycin and meropenem.

## Introduction

The emergence of antimicrobial resistance is a global problem that has been occurred both in the community setting as well as within hospitals (1, 2). It has been estimated that 50-60% of all nosocomial infections in the United States (U.S.) are due to the antibiotic resistant bacteria (3). Gram-positive bacteria-particularly gram-positive cocci like coagulase-negative Staphylococci, *Staphylococcus aureus* and *Enterococcus spp*. are extremely important pathogens in the hospital environment (4). Antimicrobial susceptibility testing is an important measure to determine the best antibiotics for the treatment of nosocomial infections. Disk diffusion test is the usual method for this purpose applied in most institutions and hospitals. Considering the value of the results of this test in decision making for antibiotic therapy of infections in hospitalized patients, it is important to know the reliability of its results. In the present study, we evaluated the reliability of resistant-reported results of disk diffusion test and determined the minimum inhibitory concentration (MIC) values for 6 routinely used antibiotics against Gram-positive microorganisms of nosocomial origin, using E-test method.

## Experimental

In a prospective Cross-sectional study performed over a 1-year period from March 2009 to March 2010 at Imam Khomeini Hospital (Tehran, Iran), clinical specimens (*e.g*. blood, tracheal secretions, wound secretions, urine, *etc*.) were obtained from hospitalized patients with nosocomial infection diagnosed using the Centers for Disease Control and Prevention/National Healthcare Safety Network (CDC/NHSN) definition of health care-associated infections (5) and sent to the hospital’s microbiology lab. All specimens underwent culture and if microbial growth occurred, differential cultures and tests were performed to identify different bacterial strains. Only aerobic Gram-positive strains underwent antimicrobial susceptibility testing using disk diffusion (Kirby-Bauer) test according to the Clinical and Laboratory Standards Institute (CLSI) guidelines (6). After the inoculation of Mueller-Hinton agar culture medium (Merck, Germany) with a direct saline suspension of isolated colonies adjusted to 0.5 McFarland turbidity standard, the antibiotic disks (Padtanteb, Iran) were placed on the agar surface. After 16-18 h of incubation at 35°C, results were interpreted as either sensitive, intermediate, or resistant according to the inhibitory zone diameters around the disks using CLSI breakpoints (7). Antibiotic disks used for the tests included: cephalothin, oxacillin, clindamycin, ciprofloxacin, vancomycin, teicoplanin (only for Enterococci), and meropenem. *Staphylococcus aureus* ATCC 29213, *Enterococcus faecalis *ATCC 29212, and *Streptococcus pneumoniae* ATCC 49619 were used as standard microorganisms for quality control of the tests.

To determine the reliability of disk diffusion test and the MIC (minimum inhibitory concentration) of applied antibiotics for bacterial isolates, E-test method was performed for all resistant isolated or intermediately sensitive to disks of any above-mentioned antibiotics using the antibiotic E-test strips (AB biomerieux, Solna, Sweden) according to the manufacturer’s guidelines. After the inoculation of Mueller-Hinton agar culture medium (blood agar for Streptococci) with a direct saline suspension of isolated colonies adjusted to 0.5 McFarland turbidity standard, E-test strips were placed on the agar surface. After 16-20 h of incubation (24 h for vancomycin and teicoplanin) at 35°C, the MIC values, where the edge of the inhibition ellipse intersects the side of the strip, were read. The results were interpreted as either sensitive, intermediate or resistant according to the MIC values using manufacturer’s breakpoints.

*Statistical analysis*

SPSS software (version 17) was used for statistical analysis. For comparing the susceptibility results between the resistant microorganisms in disk diffusion and E-test methods, chi-square and fisher exact test were used. P-value less than 0.05 was considered significant.

## Results and Discussion

During the study period, a total of 137 isolates of nosocomial Gram-positive bacteria were obtained. *Staphylococcus aureus* was the most frequently isolated microorganism (n = 77, 56.2%) followed by *Enterococcus spp.* (n = 30, 21.9%), *Staphylococcus epidermidis* (n = 21, 15.3%), *Staphylococcus hemolyticus* (n = 5, 3.6%), *Streptococcus group D* (n = 2, 1.5%), *Streptococcus pneumoniae* (n = 1, 0.7%), and *Streptococcus viridans* (n = 1, 0.7%). All of the Staphylococci were sensitive to vancomycin; therefore, vancomycin E-test was not performed for these strains.

Table 1 shows the results of E-test method for each of tested antibiotics against the isolated microorganisms along with the observed MIC values/ranges at each susceptibility level. None of ciprofloxacin- and vancomycin-resistant isolates in disk diffusion test showed sensitivity in E-test method. Significant differences between the results of disk diffusion and E-test methods were observed for clindamycin and meropenem against *S. aureus* (p = 0.01 and 0.04, respectively) and *Enterococcus spp* (p = 0.03 and 0.02, respectively). There were not any significant differences between the disk diffusion and E-test results for other microorganisms.

Periodical assessment of common microorganisms’ resistance pattern in the hospitals is essential for the selection of appropriate antibiotic regimen in patients with manifestation of an infection. Proper patient evaluation, collection of suitable patient’s biological sample and coordination with an expert clinical microbiology department help the health care workers accordingly. There are some controversies about the results of methods that were used for the assessment of microorganisms’ antimicrobial susceptibility. Disk diffusion method is the routine laboratory test for microorganisms’ antibiotic susceptibility test in our hospitals. The results of this test are reported as susceptible, intermediate resistant, or resistant. With the development of microbial resistance and change of bacterial sensitivity to antibiotics, it is important to evaluate the MIC for each pathogenic microorganism. Serial dilution or E-test stripes are used for determining MIC. Serial dilution is a precise but time consuming and personnel-dependent method.

Our data showed acceptable agreement between the microorganisms’ susceptibility results based on the disk diffusion test and the results of E-test method for cephalothin, oxacillin, ciprofloxacin, vancomycin, and teicoplanin, while there were significant differences between the results of clindamycin and meropenem. In previous studies, different levels of agreement between E-test and disk diffusion in determining antimicrobial sensitivity have been reported, depending on the types of specific organisms and antibiotics used in the studies (7-9). In a recent study performed by Erfani *et al.*, E-test method was carried out for *E.coli* strains resistant to five antibiotics in disk diffusion test. By E-test method, 47.7% of strains were sensitive to nitrofurantoin, 21.1% sensitive to gentamicin and 10.5% sensitive to cotrimoxazole, ciprofloxacin and ceftazidime (10). Therefore, it seems that the level of agreement for these two methods depends on both antibiotic and microorganism that were tested. Also, the type of applied antibiotic disks may affect the results, as the quality of disks from different manufacturers may not be similar. According to the results of antimicrobial susceptibility testing of *S.aureus, S.epidermidis* and *Enterococcus* strains, E-test method is more precise for clindamycin and meropenem. For cephalothin, oxacillin, ciprofloxacin, vancomycin, and teicoplanin, disk diffusion method has the acceptable sensitivity for the detection of resistance pattern of these microorganisms. However, it is better to use E-test method for *Stapylococcus* and *Enterococcus* strains that show resistance to either cephalothin or oxacillin disks, since the organisms with reduced (intermediate) susceptibility to these antibiotics may show resistance to disks. Moreover, due to some reports of reduced susceptibility of clinically significant Staphylococci to glycopeptide antimicrobials such as vancomycin (11, 12), vancomycin E-test can be recommended to use if any *Staphylococcus* strain show resistance to its disk. The same conclusion was made by a study that compared these two methods of vancomycin against the coagulase-negative Staphylococci isolates (CoNS) (13); in this study, four CoNS isolates were resistant to vancomycin by disk diffusion method while showing susceptibility to it by E-test method. This recommendation may also applicable for *Enterococcus* strains resistant to teicoplanin disk.

**Table1:** Results of E-test method for isolated Gram-positive bacteria resistant/intermediately resistant to antibiotics in disk diffusion test

**Antibiotic**	**Microorganism**	**Disk diffusion**	**n**	**E-test**		
				**Sensitive** **n (%) [MIC range]**	**Intermediate** **n (%) [MIC range]**	**Resistant** **n (%) [MIC range]**
Cephalothin	*S.aureus*	Resistant	46	1 (2.2) [6]	12 (26.1) [12-24]	33 (71.7) [32-256<]
	*Enterococcus*	Resistant Intermediate	29 1	1 (3.4) [4] 0	3 (10.3) [12-24] 1 (100) [16]	25 (86.2) [32-256<] 0
	*S.epidermidis*	Resistant Intermediate	6 1	1 (16.7) [2] 0	0 1 (100) [16]	5 (83.3) [24-256<] 0
	*S.hemolyticus*	Resistant	3	1 (33.3) [1]	0	2 (66.7) [256<]
	*Strep. Group D*	Resistant	1	0	0	1 (100) [256<]
Oxacillin	*S.aureus*	Resistant	49	3 (6.1) [0.12-0.38]	0	46 (93.9) [8-256<]
	*Enterococcus*	Resistant	30	1 (3.3) [4]	3 (10.0) [6-12]	26 (86.7) [48-256<]
	*S.epidermidis*	Resistant	12	0	0	12 (100) [2-256<]
	*S.hemolyticus*	Resistant	3	0	0	3 (100) [256<]
	*Strep. Group D*	Resistant	1	0	0	1 (100) [256<]
	*S.pneumoniae*	Resistant	1	0	0	1 (100) [6]
	*S.viridans*	Resistant	1	1 (100) [0.5]	0	0
Clindamycin	*S.aureus*	Resistant	49	5 (10.2) [0.02-0.64]	0	44 (89.8) [24-256<]
	*Enterococcus*	Resistant	29	5 (17.2) [0.09-12]	0	24 (82.8) [256<]
	*S.epidermidis*	Resistant	11	1 (9.1) [0.02]	1 (9.1) [2]	9 (81.8) [256<]
	*S.hemolyticus*	Resistant	3	0	0	3 (100) [256<]
	*Strep. Group D*	Resistant	1	0	0	1 (100) [256<]
	*S.pneumoniae*	Resistant	1	0	0	1 (100) [256<]
Ciprofloxacin	*S.aureus*	Resistant	46	0	0	46 (100) [5-32]
	*Enterococcus*	Resistant Intermediate	25 2	0 0	1 (4.0) [2] 2 (100) [2-3]	24 (96.0) [32<] 0
	*S.epidermidis*	Resistant	13	0	0	13 (100) [4-32<]
	*S.hemolyticus*	Resistant	3	0	0	3 (100) [32<]
	*Strep. Group D*	Resistant	1	0	0	1 (100) [32<]
Meropenem	*S.aureus*	Resistant	41	7 (17.1) [0.12-4]	12 (29.3) [5-12]	22 (53.7) [16-32<]
	*Enterococcus*	Resistant Intermediate	26 1	3 (11.5) [1-4] 0	1 (3.8) [8] 1 (100) [12]	22 (84.6) [32<] 0
	*S.epidermidis*	Resistant	11	1 (9.1) [4]	2 (18.2) [6-12]	8 (72.7) [32<]
	*S.hemolyticus*	Resistant	2	0	0	2 (100) [32<]
	*Strep. Group D*	Resistant	1	0	0	1 (100) [32<]
	*S.pneumoniae*	Resistant	1	1 (100) [0.38]	0	0
Vancomycin	*Enterococcus*	Resistant	16	0	0	16 (100) [48-256<]
	*Strep. Group D*	Resistant	1	0	0	1 (100) [256<]
Teicoplanin	*Enterococcus*	Resistant	11	1 (9.1) [4]	1 (9.1) [24]	9 (81.8) [48-256<]

MIC, minimum inhibitory concentration.

## Conclusions

In order to increase the reliability of antimicrobial susceptibility results, it is recommended to perform E-test for nosocomial Gram-positive microorganisms that show antibiotic resistance via disk diffusion test and it is more important for clindamycin and meropenem.
